# Psychosocial experiences of caring by family caregivers of patients living with prostate cancer in a teaching hospital: A descriptive phenomenological study

**DOI:** 10.1002/nop2.1869

**Published:** 2023-06-02

**Authors:** Jerry Paul K. Ninnoni, Benedicta Owoo

**Affiliations:** ^1^ School of Nursing and Midwifery, Department of Mental Health University of Cape Coast Cape Coast Ghana; ^2^ School of Nursing and Midwifery University of Cape Coast Cape Coast Ghana

**Keywords:** family caregiver, nursing, patients, prostate cancer, psychosocial

## Abstract

**Aim:**

This study explored the psychosocial experience of caregiving on the family caregiver of patients with prostate cancer in the Cape Coast metropolis of Ghana.

**Design:**

A descriptive phenomenological study was conducted through in‐depth face‐to‐face semi‐structured interviews. Twelve family caregivers of prostate cancer patients were selected through purposive sampling. Interviews were conducted until data saturation. All interviews were taped, transcribed verbatim and analysed thematically.

**Results:**

The family caregiver's psychosocial experience associated with caregiving uncovered two significant themes with 13 sub‐themes. ‘Psychological impact’ emerged as the first central theme, with anxiety, care as an obligation and feelings of inadequacy, hopelessness, uncertainty, denial and concealment as the sub‐themes. The second central theme was ‘Social impact’ with sexual concerns, role adjustment, loss of livelihood, turmoil and reduced leisure activities emerging as sub‐themes.

**Conclusion:**

The findings demonstrated that caring significantly impacts the psychological and social well‐being of the caregivers of prostate cancer patients. Therefore, there is a need for holistic assessment to include the psychosocial well‐being of family caregivers to improve quality of life. Therefore, psychiatric nurses support family caregivers through education and psychosocial interventions to improve their quality of life and enable them to care for their loved ones more effectively.

## 1 INTRODUCTION

Cancer is among the growing public health concerns globally. It is one of the chronic diseases, with mortality increasing yearly (WHO, 2021). In 2018, 18.1 million people worldwide had cancer and 9.6 million died (WHO, 2021). The International Agency for Research on Cancer (IARC) projects an estimated 28.4 million cancer cases globally by 2040, with cancer‐related deaths projected to hit 13 million (Sung et al., 2021). More than two‐thirds of the world's cancers will occur in low‐income countries (WHO, 2021). However, the most rapid increases in sub‐Saharan Africa reflect weak health infrastructure, support networks and poor survival outcomes. The low survival rate in sub‐Saharan Africa is primarily attributed to a late‐stage presentation (Jedy‐agba et al., 2016).

Among the most frequently diagnosed cancers, prostate cancer is the second leading cause of death among men globally, making it a primary global health concern (Sung et al., 2021). It was the second most frequent cancer in men and the fifth leading cause of cancer deaths in 2020 (Sung et al., 2021). Whereas high‐income countries have much higher pre‐cancer incidence rates than counterparts in less developed and poor‐resourced regions globally, the corresponding mortality rates are low (Sung et al., 2021). Sub‐Saharan Africa and, for that matter, Ghana recorded a rapid increase in the incidence rate of prostate cancer with a low survival rate (Obu, 2015). This high mortality is mainly attributed to late presentation, lack of prostate‐specific antigen (PSA) screening, ignorance and cultural beliefs (Amoah et al., 2018). These men are therefore diagnosed with the advanced form of the disease and more aggressive tumours, resulting in poor clinical outcomes. It is estimated that six in nine men develop prostate cancer in Ghana (Obiri Yeboah, 2018), and only 17.7% survive (Obu, 2015). The high incidence rate of prostate cancer is well reported in the literature (Amoako et al., 2019; Obiri Yeboah, 2018), but little is known about family caregivers' needs and concerns. For this reason, the demand for caregivers increases as the number of patients with prostate cancer increases and the condition advances.

Prostate cancer affects the men suffering from the disease and their family caregivers. In recent years, research on the impact of cancer on family caregivers has been a significant area of interest. The general pattern is that cancer imposes considerable demands upon the family caregiver's health (Kristanti, 2021; Navied et al., 2020). In addition, family members' responses to the illness can play a central role in the patient's outcomes (Barlund et al., 2021; Nemati et al., 2018). Thus, to effectively help the patient cope across the disease continuum and achieve the best quality of life, it is imperative to address the problems and psychosocial needs of the family caregiver.

Family caregivers provide most long‐care care and support to their loved ones during illness. In traditional Ghanaian society, family caregivers, which include spouses, children, mothers, fathers, siblings, friends and loved ones (Liu & Zhang, 2020), generally take responsibility for the care of the sick relation by assisting with activities of daily living. Although most caregivers take up caregiving duties willingly and feel satisfied with caring for their loved ones, it can impact their psychosocial health. These family caregivers often neglect their healthcare needs to assist their family members, deteriorating their psychosocial well‐being and subsequently affecting their quality of life (QOL) (Wong et al., 2020). Similarly, care delivery primarily focuses on the patient, often neglecting the family caregiver (Lukhmana et al., 2015). Geng et al. (2018) and Navied et al. (2020) reported a higher prevalence of anxiety and depression in cancer family caregivers, which affected their quality of life. Also, Wong et al. (2020) discovered high financial hitches among family caregivers of Chinese male patients with advanced cancer, mainly because they are the traditional breadwinners.

Several studies in Ghana have focused on patient outcomes (Amoah et al., 2018; Amoako et al., 2019). The only study examining family caregivers' experience caring for patients with prostate cancer was limited to spouses (Ofori, 2017). However, significant others are caring for patients with prostate cancer. Furthermore, understanding and identifying the psychosocial needs of the caregiver is fundamental, both for preserving the carers' quality of life and enhancing the patients' satisfaction (Abbasi et al., 2020). This further lessens the caregiver's burden, reducing distress by increasing the caregiver's motivation for the caring role (Geng et al., 2018). Given the sparse evidence on the phenomenon within the Ghanaian context, this study aimed to explore the psychosocial experiences of caring for the family caregiver of patients with prostate cancer.

## 2 METHODS

### 2.1 Study design

A descriptive phenomenological approach explored the psychosocial caregiving experience among family caregivers of prostate cancer patients. This approach led to a detailed description of family caregivers' lived experiences and identified practical ways to address the psychosocial needs of these caregivers.

### 2.2 Study setting

This study was conducted at the genito‐urinary unit of the Cape Coast Teaching Hospital. It is a referral centre for many cases in Central Ghana and beyond. In addition, the hospital is a learning centre for several schools and health professionals. It has several units to manage cancer cases, including a Genito‐urinary team. This setting was selected because the hospital is the largest referral health facility serving Central Ghana for all cancer cases. Moreover, per the researcher's knowledge of existing literature, no study on prostate cancer has been undertaken in the Central region, thus informing the choice of this setting.

### 2.3 Population

The study targeted family caregivers (spouses, parents, children, siblings, cousins, aunties, loved ones and friends) who visited the Cape Coast Teaching Hospital with their patients.

### 2.4 Inclusion criteria and exclusion criteria

As part of the inclusion criteria for the study, family caregivers who provided the most assistance to prostate cancer patients with significant experiences were selected. Expressly, these caregivers must have provided care for at least 6 months, aged ≥18 years and were willing to participate in the study with informed consent (Owoo et al., 2022). The study, however, excluded bereaved family caregivers who no longer cared for patients living with prostate cancer. In addition, patients diagnosed with benign prostatic hyperplasia were excluded from the study.

### 2.5 Sampling procedure

A purposive sampling technique was adopted to select participants with rich information about caring for patients with prostate cancer (Creswell & Creswell, 2017), with the willingness to participate and the ability to communicate experiences (Ramsook, 2018). These caregivers were recruited at the genito‐urinary clinic. Others were recruited from the surgical ward through prostate cancer patients. Twelve participants overall were recruited for the study. This was in line with Creswell's (2014) recommendation of 5–25 participants. Also, taking Glaser and Strauss (2017) emphasise saturation. The interviews were ‘saturated’ with 12 participants (Appendix S2) when no new information was uncovered (Guest et al., 2020).

## 3 DATA COLLECTION INSTRUMENT

A semi‐structured interview guide was developed based on previous research on similar areas. It used an in‐depth face‐to‐face interview approach. This method allows deep exploration of participants' thoughts and experiences in the phenomenon (Creswell, 2014). Its flexible nature allows the participants to express themselves freely as the researcher probes into areas of interest in the interview (Kusi, 2012). It also enabled direct interaction with the participants, whose responses were recorded and later cross‐checked for accuracy (Khan, 2012).

### 3.1 Pretesting the interview guide

The first two interviews were pilot interviews conducted in the same facility to test the interview procedure, guide and schedule. After the pretest, two questions under section B of the guide were merged. The question incorporated into the other was, ‘Do you know the name of the cancer your relative suffers from?’ Moreover, the inquiry into which it was merged was ‘Please kindly tell me about your relation's condition’. It was noticed that the first question was answered upon asking the second question. All individual interviews were conducted face‐to‐face by (BO) with a minimum qualification of a Master of Nursing degree.

They were also trained on both the theoretical foundation and relevant qualitative techniques.

### 3.2 Data collection and management

Initial contact was made with the prostate cancer patients in the consulting room of the genito‐urinary unit. The doctors recruited prostate cancer patients who met the inclusion criteria to the consulting room. The doctors then directed the patient to the interviewer in the consultation. Patients who agreed to participate were given information about the study. They were asked to identify their family caregiver, and their contact information was retrieved. The interviewer (BO) contacted the family caregivers on the telephone, and an explanation of the importance of the study was provided. Others were, however, met in the hospital. Participants were informed that the interviews were for research purposes only.

The interview was scheduled and conducted at convenient venues, dates and times. The interviews were held in the participants' homes, within the hospital premises at the snack bar/eatery and outside some wards. Each participant was given a consent form to read and sign/thumbprint. Those who did not have literacy were assisted, and all misunderstandings were clarified days/weeks before the interview. When participants could not read English, the interviewer (BO), fluent in English and the local language, translated into the local language (Twi) to ensure that the participants understood everything written on the consent form. The data collection lasted between March and April 2019. None of the family caregivers declined.

All information elicited was taped with participant consent and transcribed verbatim. Field notes were made during and after interviews to act as triggers for recalling the interview. These notes were used to help interpret the data, which added to the credibility of the findings. Participants were informed of further interview sessions/phone calls for clarifications when necessary. Transcription was done concurrently with the interview. Copies of the recorded interviews and transcripts were saved electronically and passworded. Hard copies of the document were labelled with pseudonyms and kept in a file separately. Informed consent forms were saved up in a locked file cabinet. Participants received no compensation for participating in this study. The interviews lasted 40–50 minutes, averaging 45 minutes.

## 4 DATA ANALYSES

Data were analysed by (BO and JPN) using Colaizzi's robust method of analysis that provides rigorous scrutiny, with each step staying close to the data. Nevertheless, the result was a concise yet all‐encompassing description of the psychosocial effect of the caregiving role on the family caregivers, validated by the participants that constructed it. Likewise, it enables the researcher to find, understand, describe and depict the experience of these family caregivers. The seven steps for the data analysis are described below.

Step 1. All recorded interviews were transcribed verbatim by (BO and JPN). Each interview took 4–5 h to be transcribed, and this was after those in the local languages (Fante and Ga) had been translated into English. This was achieved using back‐back translation while maintaining confidentiality, allowing the researcher to get a clear picture of the participant's experience. Next, the transcripts for all 12 participants were independently read repeatedly. After all the reading had been completed, a sense of ‘feel’ for what was described by the participants was achieved. Then, the researchers move on to step two.

Step 2. This step involved generating data that directly pertained to the psychosocial caregiving experience among these family caregivers. At this stage of the analysis, significant statements and phrases that pertain to the psychosocial experiences were identified and extracted from the transcribed document. Ensuring that the original meaning of the test was preserved by using participants' words.

Step 3. Formulated meanings were created from the significant statements or phrases to describe and illuminate the meanings hidden in the various contexts. Subsequently, themes were generated based on the multiple messages that conveyed similar meanings. This step moves us to step 4.

Step 4. From this process, a dominant central theme emerged ‘Psychosocial Experience’. Steps 1 to 3 were then repeated for all 12 transcripts to identify experiences in terms of the psychosocial experience familiar to all participants. Finally, the formulated meanings were categorised into two central themes and 13 subthemes based on their similarities and relationships.

Step 5. A database was created to compile all the exhaustive descriptions generated in steps 1 to 3. JPN (a Senior lecturer) reviewed the findings in terms of richness and completeness to provide sufficient explanation and confirm that the detailed description reflects the experiences the psychosocial experiences of caregiving the family caregivers.

Step 6. This step typically involves returning to research participants to check created profiles of their experiences and verify that what the researcher described accurately reflects their experience, referred to as ‘member checking’ (Lincoln & Guba, 1985). This was obtained via phone calls as participants had already been informed about future interview sessions. After this, participants were satisfied with the results, reflecting their feelings and experiences. This additional verification step further enhanced the trustworthiness of the study findings.

Step 7. Involved in incorporating any new or pertinent data obtained into the final study. However, a new theme has yet to emerge.

### 4.1 Ensuring the trustworthiness of the data

Trustworthiness or rigour of a study refers to the degree of confidence in data, interpretation and methods used to ensure the quality of a study (Stahl & King, 2020), making it worthwhile to readers (Marfo‐Amankwah, 2016). This was achieved through four main criteria: credibility, dependability, transferability and confirmability. Establishing credibility involves prolonged engagement with participants, member‐checking by returning to study participants via phone calls to check for accuracy with their experiences and reflective journaling. Procedures for achieving reliability include the maintenance of an audit trail of the process throughout the study. The reporting style of this study reflects the COREQ items of reporting qualitative studies (see Appendix S1) that provide a check for the necessary components of the design. On the other hand, confirmability was achieved by conducting member‐checking with study participants. Finally, transferability was achieved by describing participants' culture, context, selection, characteristics, data collection and analysis process.

## 5 ETHICAL CONSIDERATIONS

The authors declare that: *All methods were performed under relevant guidelines and regulations consistent with the Helsinki Declaration*. In addition, Ethical approval was granted by the Institutional Review Board (IRB) of the University of Cape Coast (UCCIRB/CHAS/2018/24) and the Ethical Committee of the Cape Coast Teaching Hospital (CCTH/RDS/2019/41). Written informed consent was obtained from participants at the beginning of the interviews after they were given information about the study and informed that they could withdraw at any time. Participants also consented to publishing the study, ensuring participant confidentiality and anonymity. With participants' permission, interviews were taped and later transcribed verbatim.

## 6 RESULTS

In the present study, all 12 participants were children and spouses of the patients (see Table 1). In addition, all 12 were Christians and Ghanaians within the Cape Coast Metropolis. Also, findings revealed that six caregivers were children of the patients, five females and one male. This finding shows that the majority of the participants were females.

**TABLE 1:** Demographic characteristics of participants.

Variable	Frequency	Percentage
Sex		
Male	1	8.3
Female	11	91.7
Age		
20–29	1	8.3
30–39	2	16.7
40–49	2	16.7
50 and above	7	58.3
Marital status		
Married	10	83.3
Not married	2	16.7
Religion		
Christianity	12	100.0
Educational level		
No formal education	2	16.7
Primary	1	8.3
High school	2	16.7
Tertiary	7	58.3
Relationship with patient		
Father	6	50.0
Husband	6	50.0
Duration of Care		
Below 12 months	1	8.3
12 to 24 months	11	91.7
Occupation		
Formal	3	25.0
Informal	7	58.3
Other (Retirees)	2	16.7

## 7 EXPERIENCE OF CAREGIVING

**FIGURE 1:**
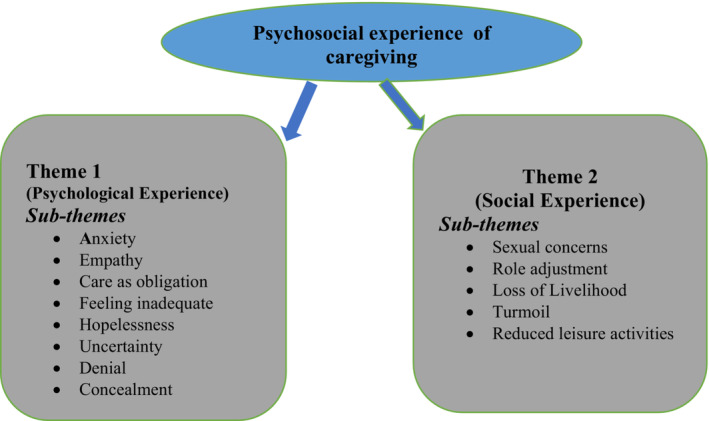
Psychological experiences of caregivers.

The ‘Psychosocial experience of caregiving’ produced two themes: ‘*psychological experiences*’ and ‘*social experiences*’. First, the participants described how the caring role affected them socially and psychologically. Under the psychological experience, participants experienced anxiety, care as an obligation/‘giving back’, and feelings of inadequacy, hopelessness, uncertainty, denial and concealment. Also, the social impact includes issues concerning sexuality, role adjustment, loss of livelihood, turmoil and reduced leisure activities (see Figure 1).

### 7.1 Anxiety

Caring for a relation with prostate cancer was described as challenging with persistent anxiety. Most participants expressed much worry in three areas, mainly due to the financial burden, changes in patients' physics and news of cancer/treatment.

Participants experienced anxiety following the news of cancer:

I was scared when I heard he has prostate cancer. My heart skipped a beat because it is a condition that kills. However, it also brings pain and debt. Mercy, married, wife

The day I heard that he has cancer, it worried me because some people say it is infectious, others also say it kills its victims within a short time. Kindness, married, wife

The participant was scared and worried upon hearing the news of cancer:

I felt someway upon hearing that he could not pass urine. Moreover, I later became more afraid when I heard he had prostate cancer because I had no idea that a condition could make someone not urinate. If you cannot urinate, it is a big problem for humans as we are. However, when I realised that something could be done about it. I was relieved. Love, married, daughter

Participants were anxious over the future financial burden:

I worry a lot about finances. Especially when it comes to settling medical bills and purchasing medications. Favour, married wife

Also, money was another major problem that made me very worried. It was our prayer that God would send help so that we could settle the medical bills and other things. Kindness, married, wife

Participants were anxious because of the patient's change in body image:

It has even turned him into an old man at this tender age, Mercy, married wife

As for worrying, hmmm, it is one thing I do best, especially when the limbs get swollen, and he starts experiencing severe pain. It gets me very worried, Humble

### 7.2 Empathy

Empathy describes the family caregiver's capacity to understand or feel what the patient is experiencing from within. For example, two participants expressed how they felt anytime they found their patients in pain and discomfort:

I also go through the same pain, Mercy, married wife

I felt pity for him because he was enduring much pain Forgive, married wife

### 7.3 Caring as an obligation/giving back

Most caregivers perceived caring as ‘Giving back/care as an obligation’. They believe these patients had cared for them in the past; thus, it is their turn and responsibility to also care for them. By so doing, they are honouring their spouses/fathers:

If I were the one in his position now, he would be the one to care for me, and I know he will gladly do it. So, if the tables have turned and he is not well, I am his child. Therefore, I must care for him, Grace, married daughter.

He has taken care of our children; we live in our house with about ten extra rooms. So, he has tried for us; I cannot leave him now, Joy, married wife

Initially, my siblings decided to hire someone to assist me, but I told them I could do the job because I knew what my dad had done for me. Thus, it is time to also care for him Hope, unmarried, son

### 7.4 I was feeling inadequate financially

Quality care depends on the caregiver's knowledge, experience and personal capability; every caregiver has a specific capacity that cannot be exceeded. The inability to provide care and satisfy the minor concerns of the patient make the caregiver feel inadequate. The participant reported that not being able to please the patient and settle medical expenses on time for treatment to commence was very worrying and frustrating:

Sometimes, being unable to raise the money for medical expenses, coupled with the old man in so much pain, is unbearable. However, they do not know what to do Favour, married wife

### 7.5 Hopelessness

Participants reported feeling hopeless, indicating that their life and plans revolved around the patient because of their caring role. Their life has come to a halt, knowing that they are trapped, and until the patient recovers, they cannot do anything about their situation:

My life has come to a standstill. I do not go anywhere. I am trapped at home. I cannot even visit my husband. My thoughts now revolve around this house: Grace, married, daughter

I have tried to speak with my siblings, but they are also not ready to help. They do not think I also have a home or life to live or that this is their father. So I do not want to talk about Love, married, daughter

Participants believe their current situation is unchangeable, and thus nothing can be done about it:

Sometimes he is very annoying; he tells you to do this while he wants something else. He irritates me a lot, but what can I do? I want to give up sometimes, but it is impossible, Joy, married wife

### 7.6 Uncertainty

The caregivers' everyday life was filled with unpredictability about the future. As a result, they felt unsure about what was expected when the patients' conditions were unpredictable.

A caregiver described the experience as follows:

Thoughts like, is, is it going to get better? Is he going to get well? These thoughts came during the earlier stages when the condition was serious, before the surgery Peace, unmarried, daughter

Apart from feeling uncertain about the future, a spouse felt that if she understood the treatment process and had been prepared for the role, she would be able to provide the support patient needed at the time:

She stated, ‘I am always worried and afraid because I do not know what this leads to anymore. More so, no one educated me on how to go about things at home, and because of that, it is a problem, as I find myself in a fix, not knowing what to do for him, especially when he is in pain’ Forgive, married, wife

### 7.7 Denial/concealment

Concealment emphasises how family caregivers suppress their natural emotions toward their patient's condition or situation. Participants avoided showing the truth and their real emotions, like tears, sadness, pain and worry, in front of the patients. Instead, participants reported that they concealed their feelings to prevent them from worrying, which helped them cope with the situation:

And even when I am worried, I hide my feelings from him because he will also become more worried Kindness, married, wife

I got tired most of the time, especially from lifting and transferring from one spot to the other with body and waist pains, but I did not want him to see that this was what I was going through. So, I will not complain about the pain, Hope, unmarried son

## 8 SOCIAL EXPERIENCE

Social impact is another theme extracted from the data that indicates the impact of caring on their day‐to‐day activities. The participants narrated their experience by reporting that they had restricted social life due to the increased burden associated with caregiving. While some participants failed to look after their children adequately, some had to give up their jobs/livelihoods. Others experienced turmoil in their marriage. As a result, sexual concerns were also significant problems for younger caregivers;

### 8.1 Sexual concerns

Participants were not open to disclosing concerns regarding sexuality. However, some spouses and children gave brief narratives regarding their poor quality of sexual life. For some family caregivers (children), the inability to sexually satisfy their spouses, girlfriend and boyfriends was because they were often away taking care of their parents. This became a source of worry for most of them. However, others (spouses of these patients) reported that the patient had no strength for sex, and some did not want to worry about them because they were sick. Others also joked with their husbands about their sexual function, even their children and nephews, while others preferred not to talk about sex:

Hmm…we only talk on the phone oo. I told you he is in a different city. So, if we only talk on the phone, that should tell you what I am trying to say Grace, unmarried daughter

And since I took up this role, my marriage has been on the rocks because I had left him and the children all alone Love, married daughter

### 8.2 Role adjustment

Participants described various roles and how some were altered due to their caring roles. Some functions were influenced by whether patients were ambulant or bedridden. For example, some spousal parts involve cooking, washing, cleaning and bathing. Also, manual lifting and handling were performed by some patients with little assistance. Other functions were reported, such as assisting the husband to the hospital for reviews, buying medication and ensuring the husband takes medication. Moreover, caregivers (children of the patient) and their families were separated from each other because they needed to care for their parents (fathers and mothers) and had their husbands and eldest children born over specific family roles, which are predominantly a ‘reserve’ of women in African traditions.

Participants (Children of the patient) had their husbands/eldest children take up the role of parenting entirely:

My children live with their dad in a different town (Mankessim). They are three, two boys and a girl. The eldest is 15 years and a boy. He assists his dad in caring for his other siblings. I visit home occasionally to check on the Love, married, daughter

Participants cleaned, bath and cooked for their patients:

When we wake up in the morning, I clean him up; then I find out that he prefers breakfast. The truth is that he could not eat much, especially when the condition became serious. I do everything from cooking, washing and cleaning for him. if he needs to go to the toilet, I must assist him. Passion, married, wife

Participants accompanied patients for reviews to ensure they had taken their medications: care involved eating, washing off their clothing and other personal hygiene needs. You can see that now he is weak, so I need to carry the water with the bucket to the bathroom for him; the primary care is eating/washing his clothing and ensuring that he takes his medications. Also, I take him on most hospital routines, especially for the reviews and investigations. Grace, married daughter

Participants were involved in lifting and turning patients without assistance:

There is no toilet in the house. Hence, I must lift him into a chamber pot and back him to the wheelchair with no one to assist. At night he may also call to help him change positions or turn in bed Love, married, daughter

### 8.3 Loss of livelihood

It was reported that working patients had saved up some money; hence, caregivers did not have to seek support elsewhere, whereas previously working caregivers had to stay home and care for their spouses and fathers. Therefore, those not working depended on their children, siblings, friends' and other family members for financial survival. The participants openly expressed their difficulties as a result of losing their livelihood. For example, a seamstress's daughter shared her experience of losing customers because she could not take the time to see them:

Yes, I was working as a seamstress, but since he took ill, most of my clients have come for their clothes. I have even cut some of them so you can imagine my heat. I cannot wait to see the Love, married, daughter

I am a farmer, but since the condition started, I have not been able to go to work. So, life has been tough Humble, married wife

### 8.4 Turmoil

Turmoil describes the confusion, agony and emotional trauma these caregivers (married daughters of patients with prostate cancer) go through in their marriages due to their role. They reported that:

And ever since I took up this role, my marriage has been on the rocks (Sad). This is a serious issue between us now (Sad). Sometimes when I call, he refuses to answer my calls. If he does, then it is all quarrels (Hmmm). Every trivial issue becomes a misunderstanding between us Love, married, daughter

He is not happy about the situation, but we both cannot do anything about it because the person in question is my dad. Because he cannot voice out his feelings or the slightest issues, he gets offended. Sometimes, even things that do not warrant that he gets offended become a major problem we are quarrelling about. We used to joke about now becoming an issue to fight about Grace, married, daughter

### 8.5 Leisure and pleasure

Leisure activities describe the time caregivers typically take after caring for the day. Participants stated that they needed more time for recreational activities. Others tried to manage some time to take care of essential things, and even that, they made sure to make it brief so they could quickly return home. Some participants, however, could make time for leisure activities anytime, any day, without worries.

Some participants manage some time to care for essential things:

yes, but it depends on the time, and it also has to be brief so that I can quickly come back home to take care of my dad. That is if I must attend an important function. Love, married, daughter

The participant could make time for recreational activities to assist the patient:

Yes, I go anywhere I want to go, especially church. There is this little girl who used to stay with us. So, if I prepare everything, I can leave. She is only there to go on errands if he needs something Kindness, married, wife

The participant spent time with friends and engaged in leisure activities at home:

yes, I am unable to live as I used to. For instance, I do not come home early when I go out, but now I do not spend more than 2 hours outside (when I am far from home) because I know I will be called. However, when I am home, I play video games, and some friends come around for us to play football. When my dad needs me, he calls on my phone. Hope, unmarried, son

Some participants, however, were able to make time for leisure activities:

Oh yes, I go anywhere I want to go. No one at home can take care of him when I leave. So, I commit him in the hands of the Lord. Joy, married, wife

However, others did not have time for recreational activities:

Now, I do not travel; I do not go anyway. Even if there is a problem in my home town, I must force to return home the same day. I cannot sleep over. This illness has affected my life badly, Mercy, the married wife.

I cannot go anywhere since the condition started, but my friends and family understand my situation. So, if I do not show up at gatherings, it is not a big problem, said Humble, married wife

## 9 DISCUSSION

### 9.1 Summary of findings from the participant's demographic data

In the present study, all 12 participants were children and spouses of the patients. All 12 were Christians and Ghanaians within the Cape Coast Metropolis. This finding is consistent with a study conducted by Ofori (2017) and the Ghana Statistical Service (2013), who reported that Christians form the largest and most popular religious group in Ghana, comprising approximately 72% of the population. This is also supported by findings from several other studies that indicated that most Ghanaian caregivers used religiosity/spirituality to deal with the strain of caregiving (Aziato et al., 2016; Ofori, 2017). Also, findings revealed that six caregivers were children of the patients, five females and one male. This finding shows that the majority of the participants were females. The results show similarities with a study conducted by Kyei‐Arthur and Atobrah (2022), who reported that most patients are cared for by females. In contrast, Sanuade and Boatemaa (2015) reported an approximately equal male‐to‐female ratio for caregivers.

Moreover, most of the participants engaged in petty trading and farming. This report validates Nyamky and Hormeku's findings (as cited in the Ghana Statistical Service, 2013), which reported that 80% of the Ghanaian populace works within the informal sector. These caregivers were also out of jobs because they needed to allow the flexibility required to work and provide care. This report is consistent with Ofori (2017) study, which reported that most participants had to stop working to care for their partners.

The psychological impact is a significant theme that emerged from the data associated with caregiving. Sub‐themes such as anxiety, care as an obligation and feeling inadequate were substantial concerns. However, hopelessness, uncertainty and concealment were other concerns encountered.

The present study reported anxiety among prostate cancer patient caregivers. Cancer is a dominant source of stress in caregivers, but several other factors may exaggerate the symptoms, such as financial burden and fear of losing a loved one. Most participants demonstrated much worry due to the financial burden, changes in patients' physic and news of cancer/treatment. The findings are consistent with Hastert et al. (2020), who reported a financial burden, which later translated into anxiety symptoms throughout the disease trajectory. Unsar et al. (2021) also said the fear of losing a loved one is a significant concern of family caregivers. This further mirrored the works of Ofori (2017), who asserted that participants were anxious about losing their husbands to prostate cancer. However, a persistently high level of anxiety in family caregivers, if not managed, may lead to depression which can negatively impact the caregiver's immune system and overall psychological health (Geng et al., 2018; Walsh et al., 2018), resulting in a disruption in the caregiving performance of the family caregiver (Saimaldaher & Wazqar, 2020). Therefore, it is essential that the family caregivers are assessed and the appropriate assistance provided.

The feeling of empathy for the patient was another theme that emerged. This describes the family caregiver's capacity to understand or feel what the patient is experiencing from within. Levenson and Ruef (1992) defined empathy as the ability to know, feel and respond appropriately to what others are feeling with the desire to relieve this suffering (as cited in Hua et al., 2021). In this study, caregivers empathised with patients, especially when they go through so much pain to pass urine. The empathetic theory proposed by Davis (2018) supports this assertion by stating that empathy‐related processes are influenced by motivating factors (e.g., the degree of suffering or deficits experienced by the affected person) and that inhibiting factors can detract from the caregiver's motivation to expend mental energy to engage in empathy toward the patient. It is, however, established that empathy and helping behaviours are hallmarks of quality care (Davis, 2018). Still, when family caregivers are challenged, caregiver confidence can be diminished, resulting in unsafe, poorly timed, and suboptimal patient care (Maguire & Maguire, 2020). Therefore, it is essential that healthcare providers become aware of the potential impact of caregiving on caregivers and assess caregiver needs, thus improving the emotional status of caregivers through education and psychosocial interventions.

The findings also revealed that family caregivers identified their role as honouring their fathers and spouses; hence, caring for them is ‘Giving back’ or an obligation to their families. Thus, they are motivated by this caring role. This assertion was supported by Rossi and Rossi (2018), who argued that individuals feel the most vital responsibility to help their parents, spouse and children, especially in times of need (Rossi & Rossi, 2018). Most participants considered this rewarding and were ready to repeat it without complaining. Comparable findings came to light in earlier studies concerned with the experiences of stroke, cancer and dementia patients. These family caregivers care for the patients at home and often do so of their own volition and genuine love for them. In such caring relationships based on passion and integrity, the caregiver derives excellent satisfaction, fulfilment and gratitude from being able to relieve, bring solace and care for the patient (Blinka et al., 2022).

The study's findings also suggest inadequacy among family caregivers of prostate cancer patients. Participants stated that their inability to provide care and satisfy the patient results from inadequacy. Others also reported that being unable to please the patient and settle medical expenses on time for treatment to commence was very worrying and frustrating. This inadequacy may be partly due to their inexperience, lack of training, financial constraints and previous encounter with care‐related problems. Shaffer et al. (2021) validate this by reporting that partners felt they could not do enough for the patients and thus continuously questioned their capacity to provide care. Again, the lack of proper information and support from healthcare professionals may have contributed to this feeling of inadequacy. A participant mentioned how unbearable it was to watch her dad in so much pain and discomfort but unable to help much. The literature has reported this observation (Kusi, Boamah Mensah, et al., 2020; Kusi, Mensah, et al., 2020). Therefore, family caregivers must be empowered with the information and skills to care for these patients at home adequately.

Participants also narrated feelings of hopelessness. Participants reported how they became helpless about their current situation and set aside personal development with their entire focus on caring for the patient. Their lives have stopped, knowing they are trapped and cannot do anything about the situation until the patient recovers.

A caregiver (daughter) explained how she could not be with her husband because she cares for her dad until he recovers. This reflects a sense of hopelessness, and these feelings appear to worsen with the cancer severity. This finding corroborates the work of Kusi, Boamah Mensah, et al. (2020); Kusi, Mensah, et al. (2020) and Ullrich et al. (2020), who validate this claim by adding that family caregivers' reported disruption in everyday life.

Similarly, the present study's findings revealed that participants were uncertain about their condition and the future. The uncertainty worried some participants, especially when they did not know how to help the patient. Sajjadi et al. (2016) defined uncertainty as the primary feeling of confusion, stress and indefiniteness associated with the disease. In addition, uncertainty is said to be a significant component of the experience of the disease that is common among participants in many studies, which causes a restriction in the caregiver's life, disrupting her psychological well‐being and affecting her adaptation to the existing situation (Nemati et al., 2018; Ainamani et al., 2020).

The findings further revealed that family caregivers had to hide their feelings in the presence of the patients for fear of making them sad and having them feel sorry for themselves. Instead, they reported trying to look strong, even in pain and intense emotion, to cope with their situation. This finding is consistent with a study by Serçe and Günüşen (2021). Moreover, they were further supported by Applebaum et al. (2021), who stated that patient relatives hid their feelings and avoided talking about the disease for fear that they might upset the patient and had difficulty coping with the patient's reactions during the treatment process. In Ghanaian society, it is argued that men are expected to exude masculine virtues of bravery, boldness, power, strength and the ability to endure physical and emotional pain, distress, agony and grief (Atobrah, 2019). This may be reflected in the participants' experiences in this study.

Applebaum and colleagues further indicated that cancer patients received the most emotional support from their close family members (Applebaum et al., 2021). In addition, their optimistic attitudes and the positive atmosphere improved patient outcomes (Laidsaar‐Powell et al., 2018). In this study, hiding emotions from patients met the family caregiver's expectation of ensuring a positive atmosphere of love. However, concealing strong feelings may harm the family caregiver's health (Sercekus et al., 2014). Therefore, it is necessary that a healthcare professional addresses this area by creating support networks where family caregivers can come together to ventilate and share their experiences.

The study also revealed several social impacts on the family caregiver. For example, the study identified sexual concerns, role adjustment, loss of livelihood, and leisure activities.

Sexuality is a stimulus for maintaining intimacy, which gives couples cohesion; this introduces the sexual concerns reported by participants. Sexuality was noted as one of the major concerns for the married children of these patients who have taken up the caregiving role. Most caregivers, especially the married children of these patients who had to leave their nuclear homes to stay with their sick fathers to care for them, had concerns about their sexual life. Although this may be strange, especially in developed countries, this observation is common among some tribes in Ghana, where daughters may return to their paternal home to care for sick parents. They were also worried that their separation from their husbands and children might lead to divorce. This assertion concurred with (Petrovic, 2018; Schulz et al., 2016), who stated that family caregivers might experience conflict among their roles and responsibilities and strained marital and family relationships. Also, the study by (Schulz et al., 2016) on the impact of caregiving on marital well‐being in adult daughters and sons revealed that these family caregivers were unhappy in their marriages due to the demands of caring (Atobrah, 2019).

Generally, Ghanaian gender ascriptions are marked by stark femininity and masculinity, attributes that feminists challenge. The ideal woman is a loving and faithful wife and mother and a ‘supplementary’ provider who submits to and respects her husband. She must be subservient, patient, and compassionate. Alternatively, hegemonic masculinity is associated with success in the economic and social spheres, characterised by the ability to provide resources, protection, defence and safety for the family, particularly female members (Atobrah, 2019; Atobrah & Ampofo, 2016). This suggests a significant imbalance in sexual life between men and women. Although daughters' marriages are threatened when they have to leave their matrimonial home to return and care for their sick parents, it is common in certain societies in Africa, including Ghana (Atobrah, 2019).

On the other hand, the current study revealed that these patients' spouses did not have these concerns; they said they prayed for their spouses to recover from the condition. More so, they have passed childbearing age and already have children; hence, there is no cause to worry about sexuality and intimacy, and they would not want to burden their sick spouses with issues regarding sex. Others also explain how they encourage them and assure them that sex is not an issue. However, Martinez et al. (2020) revealed in their study that spouses of cancer patients reported not feeling sexually attracted to their partners. This statement was, however, not evident in this study.

In contrast, Ervik et al. (2013) revealed that avoidance/inability to engage in sexual intercourse was a source of worry for caregivers. In addition, other studies reported that labelling the partner of a cancer patient as a ‘caregiver’ could have negative implications for a couple's sexual relationship, thus resulting in a loss of sexual intimacy (Martinez et al., 2020; McGillivray et al., 2022). This finding, however, is not consistent with the present study.

Role adjustment is another significant finding of this study. Most participants reported adjusting their current role to provide the necessary care. For example, participants who had children and were separated from them because they needed to care for their fathers had their husbands and eldest children responsible for managing the nuclear home in their absence. This was evident in a study that revealed that family caregivers failed to adequately care for their children because most of the attention and effort was directed toward caring for the patient (Sercekus et al., 2014). Harrison et al. (2021) further agreed by reporting negligence of spouses, children, and responsibility toward the small family by family caregivers because of the caregiving role. Nevertheless, taking up roles formally assumed by the patients resulted in role overload; this was described as a significant problem by Otis‐Green and Juarez (2012) and further affirmed by Chambers et al. (2017).

In general, Africa is a highly gendered society, and culture plays a significant part in role performance. In particular, among some cultural traditions in Ghana, women and men lived in sex‐segregated households, and women continued to live with their matrikin even after marriage (Atobrah, 2019). Children lived with their mothers on the female compound until puberty, when boys moved to live with their fathers. Such arrangements made marriage bonds weak and strengthened lineage ties. Many aspects of the social and family structures, including caring for dependent members such as the aged, the chronically sick, infants and the disabled, were sex‐segregated. Women and children did all the instrumental care of cleaning, meal preparation, and all care activities in the personal domain. Conversely, men provided necessary material provisions for maintenance (Atobrah & Ampofo, 2016).

Loss of livelihood was another primary concern of these family caregivers. Most of them had to give up on their jobs to stay home and care for the patient, as most participants were farmers and petty traders. This resulted in financial difficulties as the patients were too sick to work, and as caregivers, the burden of expensive medical treatments depended on them, thus resulting in financial pressure. This finding is consistent with the work of Nemati and colleagues, who mentioned in their study that the financial burden resulted in tension in caregiving, as the caregivers mainly were under financial pressure, thus causing them great mental anguish (Nemati et al., 2018). Several other studies reported similar results (Kusi, Boamah Mensah, et al., 2020; Sercekus et al., 2014). Moreover, the economic cost is a significant challenge among these family caregivers since patients need jobs or health insurance.

Turmoil describes the confusion, agony and emotional trauma these caregivers go through with the patients. Caregivers explained how their relationships and spouses had worsened due to the patient's condition. This made them very worried and sad; also, they feared separation/divorce from their spouses. Consistent with this study are findings from Penning et al. (2018), who reported that adult children who live with their care‐recipient parent might decline in marital satisfaction/ happiness. Another study also revealed that parents moving into the matrimonial homes of their children to be cared for might have a detrimental effect on their marriage/marital satisfaction may suffer (Jesse et al., 2018).

In addition, the study findings showed that caregivers devoted themselves entirely to caregiving and, thus, had no time for leisure and social activities. Most participants feared that the worse might happen in their absence; others also tried to make some little time to attend to pressing and vital needs. Götze et al. (2018) support this finding by arguing that caregivers face restrictions due to caring, leading to social isolation. Williams (2018) further buttresses this statement by arguing that isolation may lead to distress in the family caregiver. Other studies confirm that social isolation may negatively affect the family caregiver's well‐being (van Roij et al., 2019; Williams, 2018).

## 10 LIMITATIONS OF THE STUDY

The study may have some contextual limitations. For example, it may be necessary for the investigation to be repeated within different cultural contexts to identify and provide psychosocial support for caregivers. Also, the study focused solely on caregivers; however, including the patient perspective may lead to data triangulation and give a deeper understanding of the phenomenon. Additionally, although the strengths of phenomenological studies are well known, including the provision of unique and rich data, there are some weaknesses, bothered on subjectivity, and if ‘bracketing’ is not carefully applied, it may interfere with data interpretation. Moreover, the 12 participants were recruited from a single site, and this may have resulted in homogeneity in terms of gender and religion and thud, and recruiting from a broader and more diverse background may give a more representative picture of the phenomenon. Finally, although the researcher is a native and speaks both the local language and English fluently, certain local expressions may not have direct English translation and may influence the interpretation.

## 11 CONCLUSION

In conclusion, the result of the study demonstrates that family caregivers of prostate cancer patients have varying support needs, including social and psychological, but are often overlooked. In addition, the study has shown how participant sociodemographic variables and culture can influence the caregivers' roles and perceptions of caring. Therefore, there is a need for a holistic assessment of caregiver well‐being to improve quality of life. Therefore, healthcare professionals, including nurses, have a crucial role in the education and provision of psychosocial support tailored towards meeting the needs of different groups of caregivers. Moreover, mental health services need to be integrated into the care and support needs of patients with chronic conditions, such as prostate cancer caregivers, to promote well‐being for both the patient and the caregiver.

## 12 RELEVANCE TO CLINICAL PRACTICE

The study's findings highlighted that family caregivers' contribution towards caring for their loved ones is associated with many challenges resulting from a lack of preparation and support. Thus, clinical staff, especially nurses, should seek to identify the challenges and provide the necessary support for caregivers. For ages, healthcare providers and, most importantly, nurses have concentrated on caring for patients to neglect family caregivers. The findings of this study have revealed that these family caregivers are hidden patients themselves; hence, nurses must work closely with carers to identify their support needs. Furthermore, family caregivers need preparation to meet the demands of their new responsibilities. Nurses must therefore play their roles as resource persons with the knowledge, information and skills required to support caregivers in their caring roles. Caring may impact not only psychosocial well‐being but also physical well‐being. Therefore, some support needs may include training, for example, in moving and handling.

## AUTHOR CONTRIBUTIONS

JPN and BO contributed to the conception and design of the study. BO undertook the data collection. JPN and BO were both involved in the data analyses. JPN and BO oversaw the investigations and interpretation of results. BO drafted the initial manuscript. Both authors critically revised the manuscript's content and approved the final manuscript.

## FUNDING INFORMATION

This paper did not receive any funding.

## CONFLICT OF INTEREST STATEMENT

The authors declare no financial or financial conflict with this publication.

## RESEARCH ETHICS COMMITTEE APPROVAL

This study received approval from the Institutional Review Board (IRB) of the University of Cape Coast (UCC) (UCC1RB/CHAS/2018/24) and the Cape Coast Teaching Hospital (CCTH/RDS/2019/41). Informed consent was obtained from all participants before data collection per ethical requirements.

## Supporting information


Appendix S1

## Data Availability

All relevant data and files are included in this submission. In addition, hardcopy transcripts are available from the corresponding author on reasonable request from the School of Nursing and Midwifery via: nursing.midwifery@ucc.edu.gh/+233[03321]32440, +233[03321]32480‐9.
