# Supplementary material for: Psychosocial experiences of caring by family caregivers of patients living with prostate cancer in a teaching hospital: A descriptive phenomenological study
Source: Nurs Open. 2023 Jun 2;10(9):6268–81. doi: 10.1002/nop2.1869 (PMC10416052; doi:10.1002/nop2.1869)
Supplement: Supplementary file 1 — Appendix S1 [file NOP2-10-6268-s001.docx]

**S1 APPENDIX**

## Manuscript: Psychosocial impact of caregiving among family caregivers of patients living with prostate cancer in the Cape Coast metropolis: a descriptive phenomenological study

**Consolidated criteria for reporting qualitative studies (COREQ): a 32-item checklist (Tong, Sainsbury and Craig, 2007)**

| No. Item | Guide questions/description | Remarks | Reported on Page # |
| --- | --- | --- | --- |
| Domain 1: Research team and reﬂexivity |  |  |  |
| *Personal Characteristics* |  |  |  |
| 1. Interviewer/facilitator | Which author/s conducted the interview or focus group? | The interviews were conducted by BO | Page 6 |
| 2. Credentials | What were the researcher’s credentials? E.g. PhD, MD | The researchers’ credentials are as follows BO-  RN, BSc, Mn  JPN-BN, RN, PhD | Page 1, 6 |
| 3. Occupation | What was their occupation at the time of the study? | The researchers’ occupations are as follows  BO: Nurse  JPN: Senior Lecturer/Academic | Page 1 |
| 4. Gender | Was the researcher male or female? | The researchers’ gender are as follows  BO: female  JPN: Male | Page 1 |
| 5. Experience and training | What experience or training did the researcher have | BO- MN degree/attended a workshop on “how to conduct qualitative research”. JPN is an experienced researcher in qualitative studies and has published numerous qualitative research articles. | Page 1 |
| *Relationship with participants* |  |  |  |
| 6. Relationship established | Was a relationship established prior to study commencement? | Yes, but only for this research. | Page 6,7,8 |
| 7. Participant knowledge of the interviewer | What did the participants know about the researcher? e.g., personal goals, reasons for doing the research | \| None of the participants knew the researchers. However, all participants knew that the interview was for research purposes only. \|  \| \| --- \| --- \| | Page 6,7 |
| 8. Interviewer characteristics | What characteristics were reported about the interviewer/facilitator? e.g. Bias, assumptions, reasons and interests in the research topic | The characteristics of the researcher were that she is a practising nurse from a different hospital outside of the region where the study was conducted. | Page 6,7 |
| Domain 2: study design |  |  |  |
| *Theoretical framework* |  |  |  |
| 9. Methodological orientation and theory | What methodological orientation was stated to underpin the study? e.g. grounded theory, discourse analysis, ethnography, phenomenology, content analysis | a descriptive phenomenological approach was applied. | Page 4, 5 |
| *Participant selection* |  |  |  |
| 10. Sampling | How were the participants selected? e.g. purposive, convenience, consecutive, snowball | Purposive sampling applied | Page 5 |
| 11. Method of approach | How were participants approached? e.g. face-to-face, telephone, mail, email | They were approached face to face | Page 5,6 |
| 12. Sample size | How many participants were in the study? | There were 12 participants in all | Pages 5 and 6 |
| 13. non-participation | How many people refused to participate or dropped out? Reasons? | None | Page 6 |
| *Setting* |  |  |  |
| 14. The setting of data collection | Where was the data collected? e.g. home, clinic, workplace | Data was collected at the homes of participants, within the hospital premises, at the hospital snack bar/eatery, and outside some of the wards | Page 5, 6 |
| 15. Presence of non-participants | Was anyone else present besides the participants and researchers? | No one else was present besides the participants and the researcher | Page 6 |
| 16. Description of sample | What are the important characteristics of the sample? e.g. demographic data, date | The important characteristics of the samples were their age, gender/sex, marital status, relationship to the patient, duration of care and occupation. | Pages 9 |
| *Data collection* |  |  |  |
| 17. Interview guide | Were questions, prompts, and guides provided by the authors? Was it pilot-tested? | A guide was prepared (see …Appendix), and the pilot tested with 2 participants. | Page 6 |
| 18. Repeat interviews | Was repeat interviews carried out? If yes, how many? | No repeat interviews were carried out. | N/A |
| 19. Audio/visual recording | Did the researcher use an audio or visual recording to collect the data? | Interviews were audio recorded. | Page 6 |
| 20. Fieldnotes | Were ﬁeld notes made during and/or after the interview or focus group? | Field notes were made by BO after every interview. These field notes were used to assist in the analysis of the transcribed audio recordings. | Page 6 |
| 21. Duration | What was the duration of the interviews or focus group? | The duration of the in-depth interviews ranged from 40 to 50 minutes. | Page 7 |
| 22. Data saturation | Was the data saturation discussed? | Data saturation was discussed in the methodology section. | Page 5,6 |
| 23. Transcripts returned | Were transcripts returned to participants for comment and/or correction? | Participants were called on the phone for comments or corrections. | Page 8 |
| Domain 3: analysis and ﬁndings |  |  |  |
| *Data analysis* |  |  |  |
| 24. Number of data coders | How many data coders coded the data? | Two authors coded the data (BO, JPN | Page 7 |
|  |  |  |  |
| 25. Description of the coding tree | Did the authors provide a description of the coding tree? | A description of the coding tree has been provided in the methods. Analysis was undertaken concurrently with data collection to check for data saturation. | Page 10 |
| 26. Derivation of themes | Were themes identiﬁed in advance or derived from the data? | The themes were derived from the data during analysis. | Page 10 |
| 27. Software | What software, if applicable, was used to manage the data? | No software was used | N/A |
| 28. Participant checking | Did participants provide feedback on the ﬁndings? | Yes, participants provided feedback on the findings through phone calls. | Page 8 |
| *Reporting* |  |  |  |
| 29. Quotations presented | Were participant quotations presented to illustrate the themes/ﬁndings? Was each quotation identiﬁed? e.g. participant number | Yes, verbatim quotations were presented to illustrate the themes/ﬁndings. | Page 11-16 |
| 30. Data and ﬁndings consistent | Was there consistency between the data presented and the ﬁndings? | Yes, there was consistency between the data presented and the findings. | Page 11-22 |
| 31. Clarity of major themes | Were major themes clearly presented in the ﬁndings? | Yes, major themes were clearly presented in the findings. | Yes. they were.  Page 10 |
| 32. Clarity of minor themes | Is there a description of diverse cases or discussion of minor themes? | Yes, there was a Discussion of major and minor themes. | Page 16-21 |

**S2 APPENDIX**

**DATA COLLECTION INSTRUMENT**

**INTERVIEW GUIDE**

**SECTION A**

1. **Demographic Information**

Identification (ID.) Code ………………….

Age

Sex

Nationality

Highest educational level

Occupation

Religion

What is your relationship with the patient?

**SECTION B**

1. **What are the experiences of caregivers caring for people with prostate cancer?**

How long has your relationship been living with the condition?

How long have you been providing care for the patient?

Please kindly tell me about your relationship’s condition

What exactly do you usually do for him daily?

How do you think your caring role has affected you physically?

Probes:

Does your caring role affect your eating pattern?

How does your position affect your sleeping pattern?

Do you usually get tired?

- 1. Please tell me how you think you have been affected psychologically due to your caring role.

Probe: In terms of

Do you sometimes feel anxious?

Do you get depressed?

Do you feel distressed sometimes?

Can you discuss some of your fears with me?

Do you sometimes feel helpless?

Do you sometimes lose concentration?

Do you sometimes feel like you are not in control of the situation?

- 1. Please, how has the caring role affected your social life?

Probes:

Do you make time to groom yourself?

Do you feel Isolated?

Are you able to socialise with friends?

Are you able to make time for leisure activities?

Has your current role affected your primary role?

Has your current position affected your sex life?

- 1. In what ways has your caring role involved your work?

**SECTION C**

1. **Is there anything else you would like me to know?**
